# In vivo alterations of mitochondrial activity and amyloidosis in early-stage senescence-accelerated mice: a positron emission tomography study

**DOI:** 10.1186/s12974-021-02343-4

**Published:** 2021-12-10

**Authors:** Satoru Yamagishi, Yurika Iga, Shunsuke Ikegaya, Takeharu Kakiuchi, Hiroyuki Ohba, Shingo Nishiyama, Daisuke Fukomoto, Masakatsu Kanazawa, Norihiro Harada, Hideo Tsukada, Kohji Sato, Yasuomi Ouchi

**Affiliations:** 1 Department of Organ and Tissue Anatomy, Hamamatsu University School of Medicine, Hamamatsu, Japan; 2 Central Research Laboratory, Hamamatsu Photonics KK, Hamamatsu, Japan; 3 Department of Biofunctional Imaging, Preeminent Medical Photonics Education and Research Center, Hamamatsu University School of Medicine, 1-20-1 Handayama, Higashi-ku, Hamamatsu, 431-3192 Japan

**Keywords:** Mitochondrial activity, Senescence-accelerated prone mouse, Mild cognitive impairment, Positron emission tomography, Immunostaining

## Abstract

**Purpose:**

While marked reductions in neural activity and mitochondrial function have been reported in Alzheimer’s disease (AD), the degree of mitochondrial activity in mild cognitive impairment (MCI) or early-stage AD remains unexplored. Here, we used positron emission tomography (PET) to examine the direct relationship between mitochondrial activity (^18^F-BCPP-EF) and β-amyloid (Aβ) deposition (^11^C-PiB) in the same brains of senescence-accelerated mouse prone 10 (SAMP10) mice, an Aβ-developing neuroinflammatory animal model showing accelerated senescence with deterioration in cognitive functioning similar to that in MCI.

**Methods:**

Five- to 25-week-old SAMP10 and control SAMR1 mice, were used in the experiments. PET was used to measure the binding levels (standard uptake value ratios; SUVRs) of [^18^F]2-tert-butyl-4-chloro-5-2H-pyridazin-3-one (^18^F-BCPP-EF) for mitochondrial complex 1 availability, and ^11^C-PiB for Aβ deposition, in the same animals, and immunohistochemistry for ATPB (an ATP synthase on the mitochondrial inner membrane) was also performed, to determine changes in mitochondrial activity in relation to amyloid burden during the early stage of cognitive impairment.

**Results:**

The SUVR of ^18^F-BCPP-EF was significantly lower and that of ^11^C-PiB was higher in the 15-week-old SAMP10 mice than in the control and 5-week-old SAMP10 mice. The two parameters were found to negatively correlate with each other. The immunohistochemical analysis demonstrated temporal upregulation of ATPB levels at 15-week-old, but decreased at 25 week-old SAMP10 mice.

**Conclusion:**

The present results provide in vivo evidence of a decrease in mitochondrial energy production and elevated amyloidosis at an early stage in SAMP10 mice. The inverse correlation between these two phenomena suggests a concurrent change in neuronal energy failure by Aβ-induced elevation of neuroinflammatory responses. Comparison of PET data with histological findings suggests that temporal increase of ATPB level may not be neurofunctionally implicated during neuropathological processes, including Aβ pathology, in an animal model of early-phase AD spectrum disorder.

**Supplementary Information:**

The online version contains supplementary material available at 10.1186/s12974-021-02343-4.

## Introduction

It is well recognized that mitochondrial dysfunction contributes to the neurodegeneration occurring in Alzheimer’s disease (AD). Recent findings suggest that pathological changes that occur in AD brains, such as synaptic and neuronal losses and excessive β-amyloid (Aβ) production, may be induced by mitochondrial dysfunction and increased oxidative stress [1]. In AD patients, mitochondria are reportedly characterized by impaired functioning, including lowered oxidative phosphorylation, decreased adenosine triphosphate production (ATP), increased generation of reactive oxygen species (ROS), and compromised antioxidant defense [2]. Mild cognitive impairment (MCI) is an intermediate condition of impaired cognitive function between normal aging and dementia, and is commonly associated with progression to AD [3, 4]. The Aβ deposition rate in patients with MCI who are likely to convert to AD is greater than that in stable patients [5]. However, the in vivo relationship between mitochondrial activity and Aβ level in the state of senescence to MCI remains unclear.

We previously developed an ^18^F-BCPP-EF tracer for mitochondrial complex 1, which provides an experimental advantage in that it allows time-course changes in mitochondrial activation to be studied in vivo [6]. Specifically, this probe can visualize the availability of complex 1, the first component of four electron transport complexes in the inner mitochondrial membrane, and which can be specifically inhibited by rotenone. Using this probe, we successfully monitored dysfunction in mitochondrial activity in a rat cortical ischemia model [7] and in the parahippocampal region of the early-stage human AD brain [8].

SAMPs (senescence-accelerated mouse prones) are inbred mouse lines showing accelerated aging. There are currently nine independent strains from SAMP1 to SAMP11, which show distinct features of the aging phenomenon. Among these lines, the SAMP10 line shows neuronal loss with amyloidosis, and impairment of learning and memory due to cortical degeneration in later life [9] with age-related increase of superoxide production [10]. At 8–16 months of age, not only are the numbers of neurons reduced, but so are the lengths of dendrites and the spine densities of cortical pyramidal neurons [11]. Preceding this neuronal degeneration, microglia are affected at an earlier stage (~ 3 months-of-age) [12], with the number of segments and tips and the combined lengths of microglial processes being significantly decreased. We recently reported that at this stage, during which morphological impairments in microglia occur (i.e., the number of segments and tips and the combined lengths of microglial processes become much reduced), type 2 endocannabinoid receptor (CB2)-positive protective microglia are dominant compared with translocator protein 18 kDa (TSPO)-positive inflammatory microglia [13]. Therefore, the merit of using the SAMP10 line at this early stage is that it allows exploration of alterations in molecular events before substantial brain atrophy occurs, which is comparable with the state of the senescence in MCI in humans.

In this study, we used ^18^F-BCPP-EF and ^11^C-PiB PET tracers and immunohistochemistry to investigate the in vivo relationship between mitochondrial activity and Aβ uptake in the brains of SAMP10 mice during the early stage of neurodegeneration.

## Materials and methods

### Animals

15-week-old senescence-accelerated mouse resistant 1 (SAMR1) mice, which develop normal senescence and are often used as a control line for SAMP mice, and 5-, 15- and 25-week-old senescence-accelerated mouse prone 10 (SAMP10) mice purchased from the SLC Company (Hamamatsu, Japan), were used in this study. The mice were housed with their littermates to a maximum of five animals in each cage with food and water available ad libitum. All animal protocols and the following experiments were approved by the ethics committees of the Central Research Laboratory at Hamamatsu Photonics and Hamamatsu University School of Medicine. In addition, all applicable institutional and/or national guidelines for the care and use of animals were followed.

### Tracer production

#### PET ligand syntheses

The HM-18 cyclotron (Sumitomo Heavy Industry, Ltd., Tokyo, Japan) situated at Hamamatsu Photonics PET center was used to produce the positron-emitting radionuclides ^11^C and ^18^F in ^14^N(p,α)^11^C and ^18^O(p, n)^18^F nuclear reactions, respectively. The labeled compounds were then synthesized with a modified CUPID system (Sumitomo Heavy Industries, Ltd., Tokyo, Japan). ^18^F-BCPP-EF was radiolabeled by nucleophilic ^18^F-fluorination of the corresponding precursor, as described elsewhere [6, 7]. The radiochemical purity was more than 99% and the specific radioactivity was above 50.0 GBq/μmol. ^11^C-PiB was synthesized by *N*-methylation of the nor-compound *N*-desmethyl-PIB with ^11^C-methyl triflate [14]. The radiochemical purity was more than 96% and the specific radioactivity was above 35 GBq/μmol. Animal and human studies with ^18^FBCPP-EF reported so far guarantee that no specific off-target binding of ^18^F-BCPP-EF has been seen [15, 16].

#### PET measurements

PET was performed using a high-resolution animal PET scanner (SHR-38000, Hamamatsu Photonics, Hamamatsu, Japan) with an axial field of view (FOV) of 108 mm, a transaxial FOV of 330 mm, and a transaxial spatial resolution of 2.3 mm in the center. Animals were scanned twice a day, first with ^11^C-PiB, then 2 h later with ^18^F-BCPP-EF; the order of the tracers was not counterbalanced because carbon-11 labeled radiotracer has a 20 min half-life, indicating that a period of 5-times half-lives (100 min in this case) allows a next measurement with different PET tracer because a radioactivity once injected into the body is theoretically nearly negligible. The use of double tracers with carbon-11 and fluorine-18 positron emitters enabled two scans in the same animal without delay. One limitation here, however, is that we cannot exclude a possibility that the molecule once injected could still bind without emitting a PET signal and compete with the next tracer for potential targets. Isoflurane at 1.5–2.0% in oxygen was used to anesthetize the mice for the duration of the entire scans. The animals were placed in the prone position on a fixation plate and were then set within the gantry hole of the PET scanner. After a 15-min transmission scan using an external ^68^Ge/^68^ Ga rod source (67 MBq) for attenuation correction, serial emission scans lasting for 70 min and 80 min were performed immediately following injections of ^11^C-PiB at a dose of 8 MBq and ^18^F-BCPP-EF at a dose of 5 MBq, respectively. The tracers were injected intravenously through a cannula inserted into the tail vein. No arterial sampling was conducted. The PET data were reconstructed using 3D DRAMA (iteration 2, gamma 0.1) with a Gaussian filter of 1.0 mm full-width at half-maximum (FWHM), yielding a voxel size of 0.65 × 0.65 × 1.0167 mm for the reconstructed images. To obtain anatomical information, X-ray CT scans were performed immediately following the PET measurements, using a ClairvivoCT (Shimadzu Corporation, Kyoto, Japan).

### Immunohistochemistry

Immunostaining was performed as previously reported [17]. Briefly, mice were anesthetized with chloral hydrate (400 mg/kg) and transcardially perfused with phosphate-buffered saline (PBS) followed by 4% paraformaldehyde (PFA; pH 7.4). Their brains were removed, post-fixed in 4% PFA, and immersed in 30% sucrose in PBS as cryoprotectant until the tissue sank. Tissues were then frozen in dry ice and stored at − 80 °C. Frozen coronal sections of 20-µm thickness were cut using a cryostat. The slides were blocked with 10% donkey serum in PBS containing 0.1% Triton X-100 for 1 h at room temperature (RT), followed by incubation overnight at 4 °C with primary antibodies. After washing with PBS for three times, the slides were incubated for 1 h at RT with fluorescent conjugated secondary antibodies. Then, they were washed three times with PBS and stained with DAPI to visualize nuclei. Fluorescent images of a single focal plane were obtained by confocal microscopy using a 63 × lens (SP8, Leica, Wetzlar, Germany). The images were binarized and quantified using Fiji software. The following primary antibodies were used in this study: mouse anti-ATPB (1:500, Abcam, Tokyo, Japan), rabbit anti-ATPB (1:500, Proteintech, Rosemont, USA), rabbit anti-CaMKII (1:500, Abcam), rabbit anti-GFAP (1:500, DAKO/Agilent, Santa Clara, USA), rabbit anti-Iba1 (1:500, WAKO, Saitama, Japan), rat anti-TREM2 (1:500, Abcam), rabbit anti-amyloid beta (1:500, Abcam), rat anti-CD31 (1:100, BD Biosciences, Franklin Lakes, USA) and rat anti-PDGFRβ (1:250, kindly provided by Prof. Takakura, Osaka University). The secondary antibodies were as follows: Alexa Fluor 488 anti-rabbit IgG and anti-mouse IgG, Alexa Fluor 568 anti-rabbit IgG, Alexa Fluor 594 anti-mouse IgG, and Alexa Fluor 647 anti-goat IgG (1:500, Thermo Fisher Scientific, Waltham, USA).

### Data and statistical analyses

The PET data were analyzed with PMOD image software (version 3.7; PMOD Technologies Ltd, Zurich, Switzerland). The SUVRs for ^11^C-PiB and ^18^F-BCPP-EF were estimated by dividing the target SUV by the cerebellar SUV, with the latter being taken to indicate the background level (the cerebellar cortex being chosen as a reference region) [8, 18]. The SUVs were calculated as the measured radioactivity divided by the ratio of the total injected dose to the mouse body weight. As described elsewhere [13, 19], ellipsoid volume of interest (nearly 2.9 mm × 0.8 mm × 1.5 mm in diameter) ranging from 14 to 16 mm^3^ were placed over the frontal cortex anteriorly under the Bregma by referring to the X-ray CT images [13] (Additional file 1: Fig. S1). One-way analysis of variance (ANOVA) was performed to compare tracer uptake and mouse age, with the significance level set at *p* < 0.05 with a correction for multiple comparisons (Bonferroni test). Within each age group, correlation analysis was performed between the two tracer SUVRs (^11^C-PiB SUVR and ^18^F-BCPP-EF SUVR, at either 5 or 15 weeks of age) using the false discovery rate (FDR) correction for multiple correlations (*p* < 0.05), to examine deviations in patterns of the parameters in the living brains of SAMP10 mice in relation to the progression of senescence.

## Results

### PET findings

**Fig.1:**
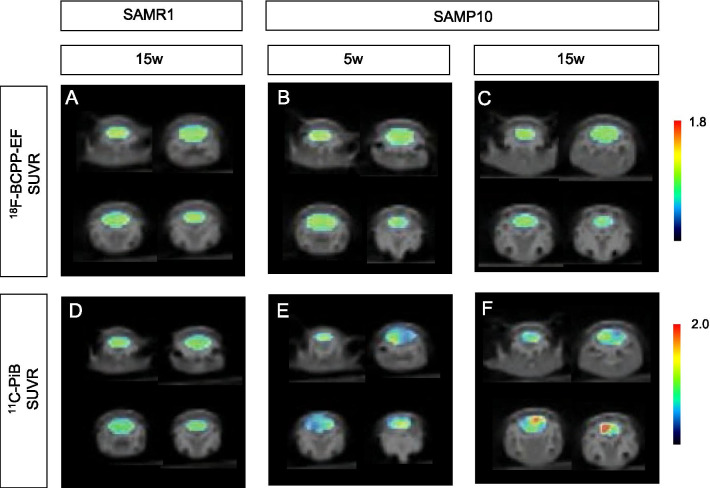
Parametric PET images of ^18^F-BCPP-EF (upper panels) and ^11^C-PiB (lower panels) tracers in 15-week-old SAMR10 mice (**A**, **D**), and 5-week-old (**B**, **E**) and 15-week-old (**C**, **F**) SAMP10 mice. The PET data are superimposed on X-ray CT images, and the color bar denotes the SUVR

**Fig. 2:**
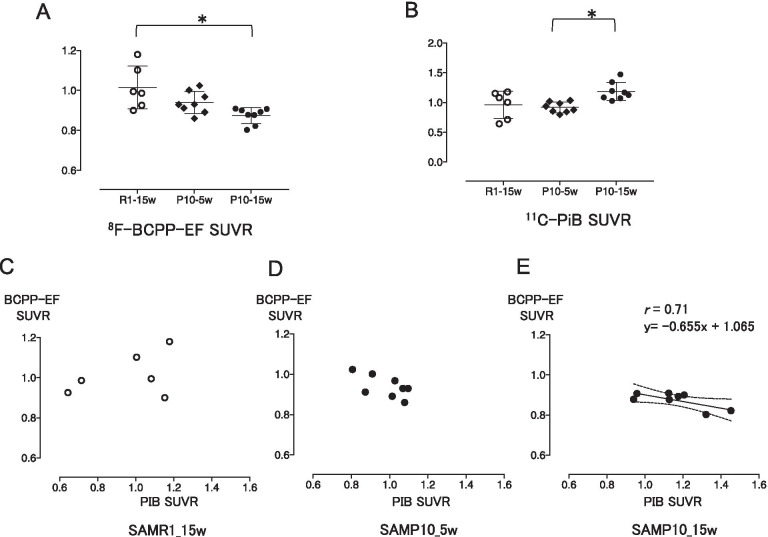
The SUVRs of ^18^F-BCPP-EF (**A**) and ^11^C-PiB (**B**) tracers and the relationships between them (**C**–**E**). The SUVR of ^18^F-BCPP-EF was significantly lower, and that of ^11^C-PiB (B) was higher in the 15-week-old SAMP10 mice than in the controls (**A** and **B**, *p < 0.05). A negative correlation between the binding of the two tracers was found in the 15-week-old SAMP10 mice (**E**). The dotted lines in E represent the 95% confidence intervals for the correlation. *n* = 6 for 15-week-old SAMR1 mice and *n* = 8 for 5-week-old and 15-week-old SAMP10 mice

We first analyzed the mitochondrial activity in the brains of SAMR1 mice at 15 weeks of age and SAMP10 mice at 5 and 15 weeks of age using ^18^F-BCPP-EF. Figure 1 shows the parametric PET images of ^18^F-BCPP-EF uptake superimposed on CT images of 15-week-old SAMR1 mice (A) and 5-week-old (B) and 15-week-old (C) SAMP10 mice. The SUVRs of ^18^F-BCPP-EF in the brain did not show a significant difference between SAMR1 mice and 5-week-old SAMP10 mice (Table 1). By contrast, in 15-week-old SAMP10 mice, the SUVR was lower throughout the brain (Fig. 1C), being significantly lower than that of the 15-week-old SAMR1 mice (*p* = 0.0036, Fig. 2A), meaning that mitochondrial oxidative metabolism had decreased in the SAMP10 mice around the period of 15 weeks of age. Next, we analyzed the Aβ level using ^11^C-PiB. The SUVRs of the control SAMR1 mice and 5-week-old SAMP10 mice did not show a significant difference (Figs. 1D, E, 2B). By contrast, the 15-week-old SAMP10 mice (Fig. 1F) showed a significantly higher ^11^C-PiB SUVR than the 5-week-old mice (Fig. 1E), indicating that Aβ accumulation is detectable by PET at around 15 weeks of age. The lack of a significant difference between the 15-week-old SAMP10 and SAMR1 mice was due to a large variation in the ^11^C-PiB SUVR in the SAMR1 mice. Direct comparisons between the ^18^F-BCPP-EF and ^11^C-PiB SUVRs showed a significant negative correlation in 15-week-old SAMP10 mice, but no significant correlation in 15-week-old SAMR1 mice and a tendency of correlation (*p* = 0.133, *r* = 0.578, *y* = − 0.32*x* + 1.18) in 5-week-old SAMP10. This finding indicates that a greater Aβ appearance reduces mitochondrial availability in 15-week-old SAMP10 mice, and vice versa.

**Table 1:** Differences in the levels of SUVR of ^18^F-BCPP-EF and ^11^C-PiB in the cortex

Group	Week	^18^F-BCPP-EF	^11^C-PiB
SAMP10	5	0.94 ± 0.06	0.95 ± 0.09
	15	0.87 ± 0.04*	1.187 ± 0.15**
SAMR1	15	1.01 ± 0.11	0.96 ± 0.23

^*^p < 0.05 vs SAMR1 (corrected with Bonferroni test)

^**^p < 0.05 vs SAMP10 at 5 weeks (corrected with Bonferroni test)

### Immunohistochemical findings

**Fig. 3:**
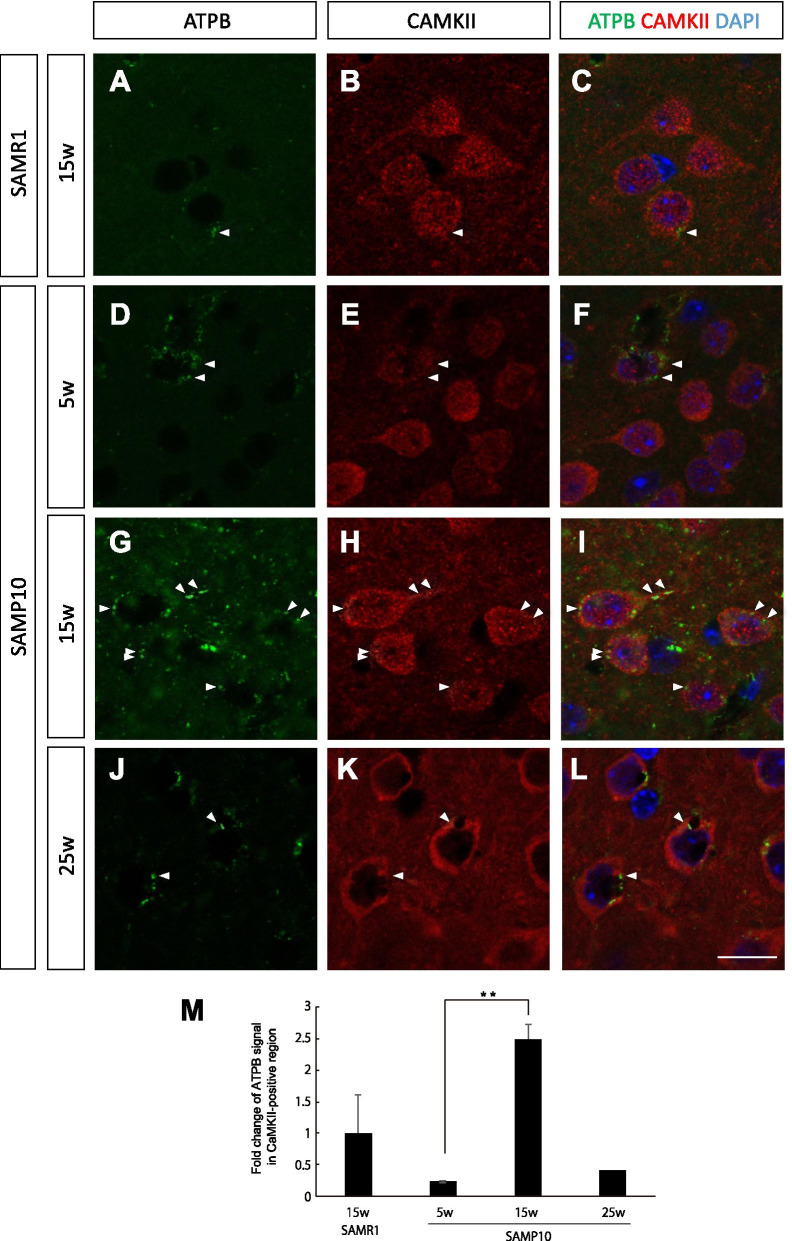
Double immunostaining for ATPB (green) and CaMKII (red) in the cerebral cortex of 15-week-old SAMR1 mice (**A**–**C**) and 5-week-old (**D**–**F**), 15-week-old (**G**–**I**) and 25-week-old (**J**–**L**) SAMP10 mice. **M** Quantification of the immunofluorescent signals. Note that the ATPB signal had greatly increased and was localized to the neurons at 15 weeks of age, but dramatically decreased at 25-week-old. Arrowheads indicate ATPB signals. Scale bar: 10 μm. Data are means ± SEs. They were analyzed by one-way ANOVA followed by the Bonferroni test (**p < 0.01). *n* = 4 for each group, except for 25-week-old SAMP10 mice (*n* = 2)

According to the PET finding of decreased mitochondrial activity at 15 weeks of age in SAMP10 mice, we next examined the expression level of ATPB, a component of ATP synthase on the mitochondrial inner membrane, by immunohistochemical analyses. The specificity of monoclonal anti-ATPB antibody was verified by another polyclonal antibody (Additional file 1: Fig. S2). In the control mice (15-week-old SAMR1 mice and 5-week-old SAMP10 mice), there were a small number of ATPB-positive signals in the soma of neurons in the cerebral cortex (Fig. 3A–F). Interestingly, in the 15-week-old SAMP10 mice, the intensity and numbers of ATPB-positive signals were dramatically elevated (Fig. 3G–I), which was opposite to the ^18^F-BCPP-EF PET finding. One possibility of this discrepancy is that there might be a difference in expression between MC-1 and MC-V, the latter of which directly engages in ATP production. The immuno-positive punctuate signals were observed not only in the soma, but also in neurites. This result suggests that neurons are likely to increase ATP production in the early stages of neurodegeneration, but not successful. In the 25-week-old SAMP10 mice, the level of ATPB was decreased to basal level (Fig. 3J–L).

**Fig. 4:**
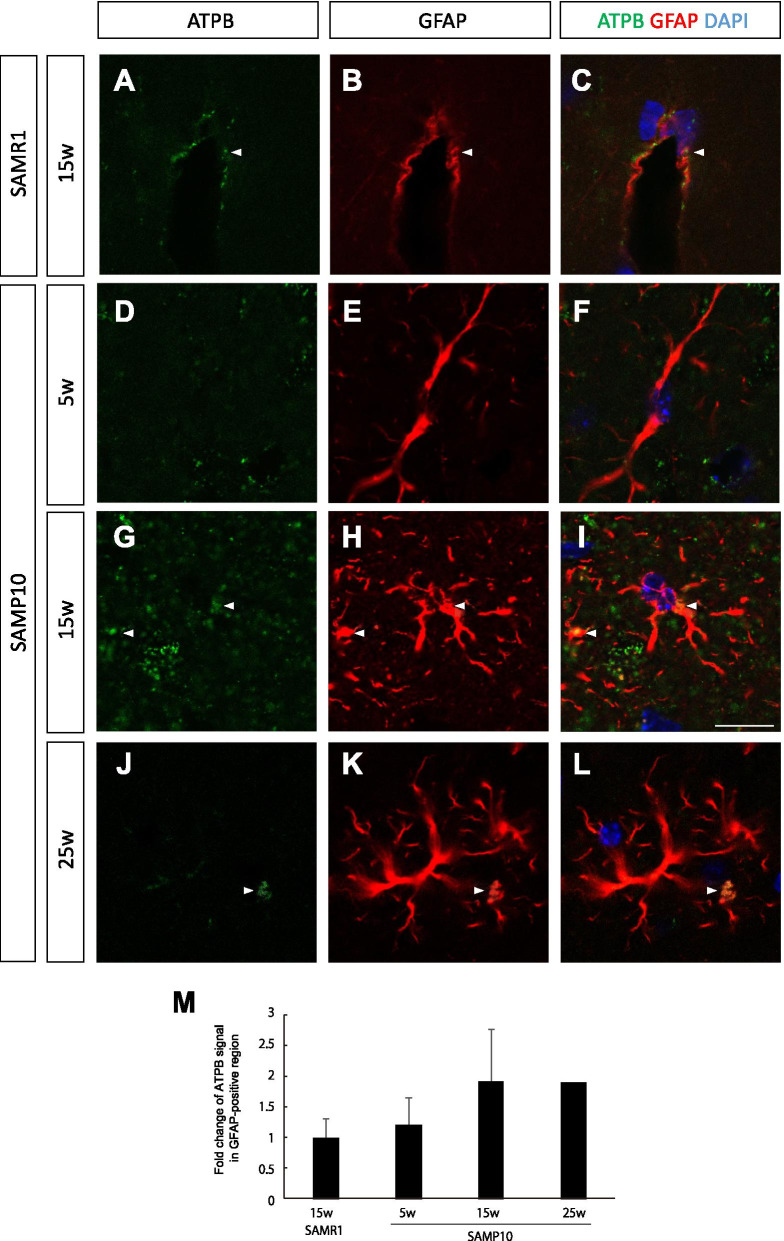
Immunostaining for ATPB (green) and GFAP (red) in the cerebral cortex of 15-week-old SAMR1 mice (**A**–**C**) and 5-week-old (**D**–**F**), 15-week-old (**G**–**I**) and 25-week-old (**J**–**L**) SAMP10 mice. (M) Quantification of the immunofluorescent signals. Note that both ATPB and GFAP immuno-reactivity were much higher in the 15-week-old SAMP10 mice than in the two control groups. Although GFAP immuno-reactivity stays higher in the 25-week-old SAMP10 mice, ATPB signal was dramatically reduced to the basal level. The localization of ATPB was observed in GFAP+ astrocytes. Arrowheads indicate ATPB signals. Scale bar: 10 μm. Data are means ± SEs. *n* = 4 for each group, except for 25-week-old SAMP10 mice (*n* = 2)

As ATPB immuno-positive signals were also observed in non-neuronal cells, as shown in Fig. 3G, we next analyzed the localization of ATPB in astrocytes. In 15-week-old SAMR1 mice, the GFAP signal was mainly observed in the endfeet of perivascular astrocytes in the cerebral cortex (Fig. 4A–C), where we also found that ATPB was localized. While the ATPB immuno-positive signal was very low in 5-week-old SAMP10 mice (Fig. 4D–F), both the ATPB signal and the overall GFAP signal were much higher throughout the cerebral cortex in the 15-week-old SAMP10 mice, indicating proliferation of reactive astrocytes consistent with previous reports (Fig. 4G–I) [20]. As expected, the ATPB signal was colocalized within the GFAP+ astrocytes. Further analysis of the upregulation of ATPB in pericytes was performed, because many ATPB immuno-positive signals were found along a capillary (Additional file 1: Fig. S3), and progressive pericyte loss in a mouse model of AD pathogenesis was previously reported [21]. However, most of the ATPB signals along the capillary were located in astrocytes, not in pericytes (Additional file 1: Fig. S2).

**Fig. 5:**
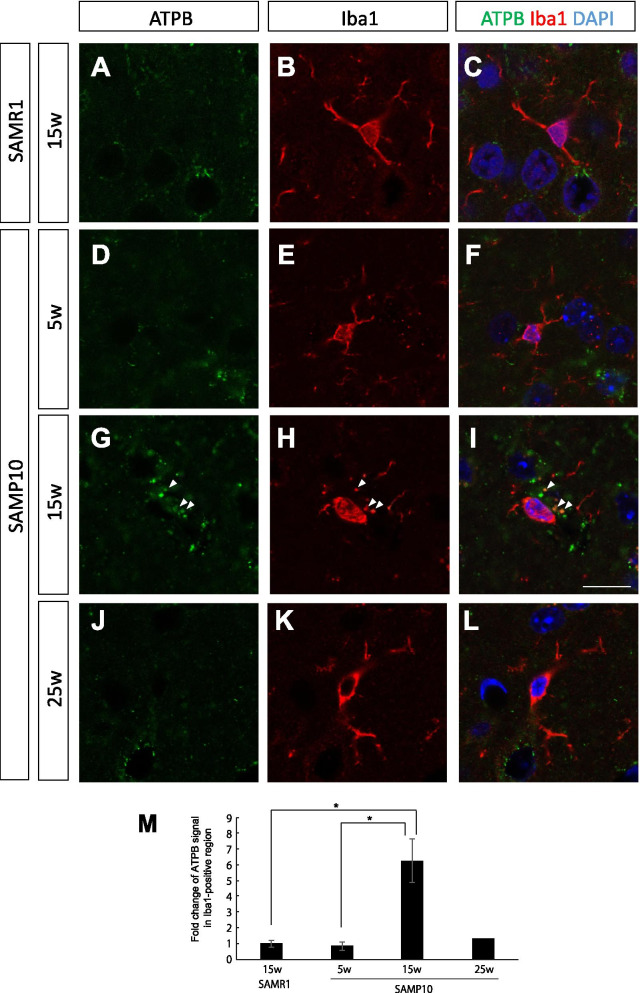
Double immunostaining for ATPB (green) and Iba1 (red) in the cerebral cortex of 15-week-old SAMR1 mice (**A**–**C**) and 5-week-old (**D**–**F**), 15-week-old (**G**–**I**) and 25-week-old (**J**–**L**) SAMP10 mice. (M) Quantification of the immunofluorescent signals. Note that the ATPB signal was much higher and localized to the microglia in the 15-week-old SAMP10 mice, but not at the 25-week-old. Arrowheads indicate ATPB signals. Scale bar: 10 μm. Data are means ± SEs. They were analyzed by one-way ANOVA followed by the Bonferroni test (*p < 0.05). *n* = 4 for each group, except for 25-week-old SAMP10 mice (*n* = 2)

**Fig. 6:**
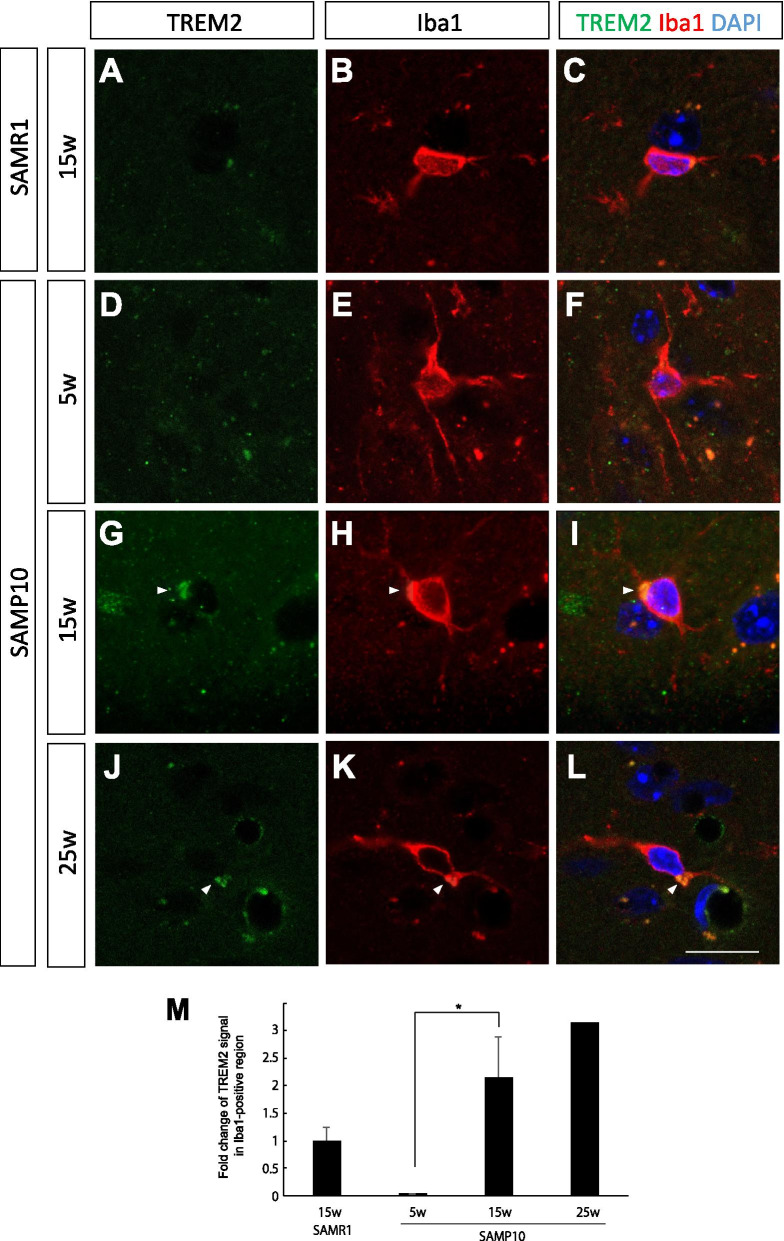
Immunostaining for TREM2 (green) and Iba1 (red) in the cerebral cortex of 15-week-old SAMR1 mice (**A**–**C**) and 5-week-old (**D**–**F**), 15-week-old (**G**–**I**) and 25-week-old (**J**–**L**) SAMP10 mice. **M** Quantification of the immunofluorescent signals. Note that TREM2 immuno-reactivity was higher and localized to the microglia in the 15- and 25-week-old SAMP10 mice. Arrowheads indicate TREM2 signals. Scale bar: 10 μm. Data are means ± SEs. They were analyzed by one-way ANOVA followed by the Bonferroni test (*p < 0.05). n = 4 for each group, except for 25-week-old SAMP10 mice (*n* = 2)

Figure 5 shows the results of investigations into whether ATPB was localized to microglia. In the control mice (15-week-old SAMR1 and 5-week-old SAMP10 mice), the intensities of the ATPB immuno-positive signals were too weak to detect in Iba1+ microglia. By contrast, a detectable level of ATPB-positive signal was colocalized with Iba1+ microglia in the 15-week-old SAMP10 mice. The microglia in these 15-week-old SAMP10 mice may have been protective microglia, as we recently reported that CB2+ microglia activation was dominant at an early stage of neurodegeneration [13]. In the 25-week-old SAMP10 mice, ATPB-positive signal was disappeared from Iba1+ microglia. Next, we analyzed the expression of TREM2, which reportedly has a protective function to prevent AD progression [22, 23]. While the TREM2 immuno-positive signal was very low in the microglia in the control mice (Fig. 6A–F), intensive TREM2 immuno-positive punctuate signals were observed in Iba1+ microglia in 15- and 25-week-old SAMP10 mice (Fig. 6G–L). This result supports our previous finding of the activation of protective microglia in SAMP10 mice at this early stage, which is comparable to the state of senescence in early-stage MCI in humans.

**Fig. 7:**
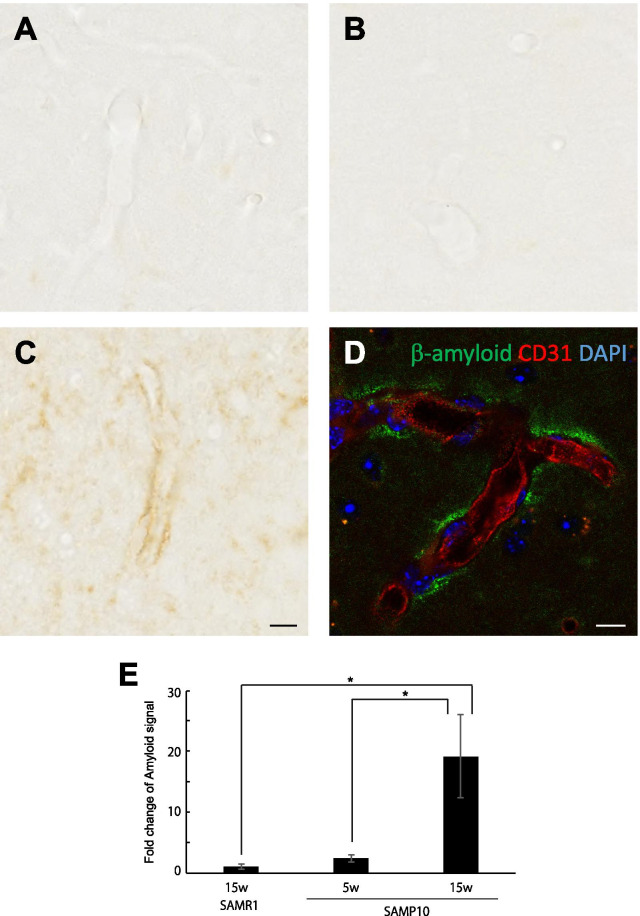
Immunostaining for β-amyloid in the cerebral cortex of 15-week-old SAMR1 mice (**A**) and 5-week-old (**B**) and 15-week-old (**C**, **D**) SAMP10 mice. **D** Section was stained for β-amyloid (green), CD31 (red) and DAPI (blue). It is worth noting that immunoreactivity of β-amyloid was observed in the perivascular space in the 15-week-old SAMP10 mice. Senile plaque was not observed. Scale bars: 10 μm. **E** Quantification of the immunostainings. Data are means ± SEs. They were analyzed by one-way ANOVA followed by the Bonferroni test (*p < 0.05). *n* = 4 for each group

Lastly, we immunohistochemically analyzed amyloid deposition. As expected from PET analysis, we observed intensive immunoreactivity against beta-amyloid in 15-week-old SAMP10 mice, but not in 15-week-old SAMR1 mice and younger SAMP10 mice (Fig. 7). The immunoreactivity was detected at perivascular region, suggesting clearance of β-amyloid from perivascular drainage system. Senile plaque was not detected as previously reported [24].

## Discussion

### Detection of mitochondrial activity and amyloid deposition in SAMP10 mice

In the PET study, we showed that the lower SUVRs of ^18^F-BCPP-EF and the higher SUVRs of ^11^C-PiB in the cerebral cortex in 15-week-old SAMP10 mice than in control mice, and that these SUVRs were inversely correlated with each other in 15-week-old SAMP10 mice (Figs. 1, 2). To our knowledge, this is the first study to report a change in mitochondrial activity in SAMP10 mice on in vivo PET imaging, highlighting decreased mitochondrial activity at an early stage in SAMP10 mice and immunohistochemical dynamic change of expression level of ATPB depending on the progression of pathology, comparable to the early stage of AD spectrum disorder in humans. Although many researchers have investigated frontal lobe atrophy and Aβ-deposition using SAMP10 mice older than 7 months, we here used younger mice to analyze early molecular changes at the beginning of neurodegeneration. Aβ-deposition was not obvious at 5 weeks of age, but we did observe an elevation in the SUVR of ^11^C-PiB in 15-week-old SAMP10 mice. In contrast to the decreased SUVR of ^18^F-BCPP-EF, which reflects oxidative phosphorylation in mitochondria, at the same stage (Figs. 1, 2), we could observe intensive immuno-reactivity of ATPB. It is probable that the component of metabolism had increased to try to compensate for an energy loss in neurons, which happens with mitochondrial dysfunction in the pathological condition of Aβ-deposition in the AD brain, but unsuccessful. In a later stage of disease, further reduced energy production and increased superoxide generation would occur in association with more serious pathological events, as neurodegeneration progresses [10, 25, 26]*.*

### A possible key player: glial cells

It is well-known that glial cells play important roles in the degenerated brain, serving as main players in neuroinflammation. In the present study, we found the inverse correlation between Aβ uptake and mitochondrial activity only in 15-week-old SAMP10. Considering the neuroprotective glial activity especially at an early stage of neurodegeneration, a lack of correlation seen in 5-week-old SAMP10 might be ascribed to greater activation of neuroprotective glial cells as suggested in our recent study [13]. Aβ appearance affects neurons and neuroinflammatory cells, and upregulation of IL-1β and IFN-β in 3-month-old SAMP10 mice, and IL-6 in later-stage SAMP10 mice, have been observed [27]. In our recent study, neuroprotective microglia were more dominant than neuroinflammatory microglia in the early stage of neurodegeneration in SAMP10 mice [13]. Although the high energy requirements of neurons mean that they form a major contribution to the oxidative metabolism of the brain, glial cells are also responsible for some of the oxidative metabolism. The oxidative metabolism in microglia changes as symptoms vary. While induction of M1-type inflammatory microglia by lipopolysaccharide leads to a reduction of mitochondrial oxygen consumption and lactate production, these reductions are not caused by IL-4/IL-13, inducers of M2-type protective microglia [28]. This indicates the occurrence of higher metabolism in M2-type microglia. When protective microglia are dominant in the early stage of neurodegeneration, oxidative phosphorylation in mitochondria remains at a high level. However, as the number of protective microglia decreases and neuroinflammatory microglia become prominent with the progression of Aβ deposition in later stages, the oxidative phosphorylation activity in microglia will be reduced by mitochondrial dysfunction, concomitant with that in neurons [28].

### Interaction of amyloid and neuronal mitochondria

One of the mechanisms by which amyloid leads to mitochondrial dysfunction is the transportation of APP into mitochondria [29]. Pre-sequence protein (PreP), a processing enzyme that recognizes mitochondrial-targeting signal peptides and cleaves after protein import, can degrade Aβ in the mitochondria [30]. Interestingly, the proteolytic activity of PreP is decreased in the AD brain [31]. Aβ can be localized to the inner mitochondrial membrane [32], and constituents of the γ-secretase complex, such as nicastrin, APH-1, PEN-2, and presenilin-1, which function in APP processing, are also localized within the mitochondria-associated membrane [33, 34]. It has been reported that mitochondrial dysfunction and neurodegeneration occur in model mice with deleted HtrA2, a serine protease that interacts with Aβ, APP, and presenilin-1 within the intermembrane space [35]. These pieces of evidence suggest that Aβ and Aβ-related enzymes are linked with failure of mitochondrial function.

### Immunohistochemical analyses

Our immunohistochemical analyses presented in Figs. 3, 4 and 5 show that in 15-week-old SAMP10 mice, mitochondrial ATPB, a key enzyme for ATP production, was mainly present in neurons, although some was present in microglia, and slightly elevated levels were also present in reactive/perivascular astrocytes. Because the population of neurons was most abundant in the cerebral cortex, the primary contributor to the SUVR of ^18^F-BCPP-EF was considered to be neurons. As the number of GFAP+ reactive astrocytes in the cortex had increased in the 15-week-old SAMP10 mice [20], and specific CB2+ protective microglia were activated at this early stage in the SAMP10 mice [13], we speculate that the extent of the polarized neuroinflammatory responses (neurotoxic or neuroprotective) of these glial cells would be of relevance to future neuronal degeneration. The protective cytokines (including neurotrophic factors) that are released from microglia exposed to neuropathic substances such as Aβ might stimulate neurons to supply more glucose and glutamine from perivascular astrocytes. These supplies may enable neurons to survive by temporarily increasing energy production.

Normally Aβ clearance is mediated by perivascular drainage systems, which is depending on ApoE isoforms [36–39]. The blood–brain barrier breakdown caused by pericyte dysfunction and impairment of platelet-derived growth factor receptor-β (PDGFRβ) signaling have recently been attracting attention as pathological features of AD [36, 40]. Pericytes are involved in the efflux of accumulated Aβ in the brain [41]. Originally, we thought that pericyte activity might be elevated at an early stage of neurodegeneration; however, in 15-week-old SAMP10 mice, there was no increase in the immunostaining level of ATPB in pericytes, while adjacent astrocytes contained a higher level of ATPB (Additional file 1: Fig. S2). In this relatively early stage animal model, the contribution of pericytes to accumulation of Aβ in the brain parenchyma may be minimal. Indeed, the number of pericytes starts to decrease after 4 months of age in 5xFAD mice [21].

We also observed elevation of TREM2 (an important protein for clearance of Aβ) in microglia in 15- and 25-week-old SAMP10 mice (Fig. 6). TREM2 is a key player in the switching of microglia from a homeostatic state to a disease-associated state. Interestingly, soluble TREM2 in cerebrospinal fluid is higher in Aβ+ Tau+ MCI patients than in CN individuals [42]. Furthermore, TREM2 expression in mononuclear cells in the peripheral blood of MCI patients, especially those likely to convert to AD, was significantly higher than in CN individuals [43]. TREM2 activates the mTOR pathway that regulates mitochondrial energy production by promoting the synthesis of mitochondrial proteins, including components of MC-1 and MC-5 [44]. Therefore, the correlation between mitochondrial activity and TREM2 expression in the 15- and 25-week-old SAMP10 mice is easily speculated.

## Conclusion

In this study, we found significant reduction in SUVR of ^18^F-BCPP-EF and increase in ^11^C-PiB SUVR in the brains of 15-week-old SAMP10 mice compared with 5-week-old SAMP10 mice and 15-week-old SAMR1 mice, indicating a decrease in mitochondrial activity and elevated amyloidosis occurring in the brain in the early state of senescence towards cognitive impairment and AD spectrum-type neurodegeneration. Interestingly, the SUVR of ^18^F-BCPP-EF was negatively correlated with that of ^11^C-PiB. We also found temporal upregulation of ATPB followed by strong downregulation during progression of neurodegeneration, mainly in the neurons in the cerebral cortex of SAMP10 mice. Hence, Aβ-induced neuroinflammation may shift the net production of ATP from neuronal oxidative metabolism to anaerobic glycolysis, as supported by the negative correlation between mitochondrial activity and Aβ appearance. This contention could lead to a therapeutic expectation that a specific treatment to sustain mitochondrial activity might help ameliorate senescence-related neuroinflammation and degeneration.

## Limitations

There are several limitations of note in the current study. First, although we measured changes in the uptake of both ^18^F-BCPP-EF and ^11^C-PiB in 5- and 15-week-old SAMP10 mice and control SAMR1 mice, the PET data did not reveal the mitochondrial activities of any particular cell types: neurons, microglia, oligodendrocytes and/or astrocytes. As the present study focused on changes in early-stage SAMP10 mice, observations at a later stage might reveal different expression of inflammatory substances and cells; hence, a further study is needed to address this issue in the broader time course of senescence-related neuronal loss. Second, because the spatial resolution of the PET scanner used was only 2.3 mm, the results may be subject to partial volume effects. To reduce such effects, we tried to set the measurement VOIs to at least twice the size of the FWHM of the scanner. This limitation did not allow to analyze PET data regionally in the mouse brain. An alternative would be to use autoradiography instead of PET, although measures of parameter change within the same animal cannot be obtained using autoradiography. Third, as we did not make additional PET measurements using a tracer for neuroinflammation in the same animals, we could not evaluate the direct contribution of neuroinflammation to the change in mitochondrial activity. However, taking our recent PET results on neuroinflammation [13] into consideration, we consider an in vivo relationship to be likely, as mentioned above. Lastly, we cannot conclude whether our observation is applicable to other AD model mice or human patients. Further studies using ^18^F-BCPP-EF are needed to address the issues.

## Supplementary Information

**Additional file 1: Figure S1** The location of volume of interest. The VOI is displaced three-dimensionally on CT and the corresponding PET images. **Fig. S2** Double immunostaining for ATPB by mouse monoclonal (A, green) and rabbit polyclonal (B, red) antibodies in the cerebral cortex of 15-week-old SAMP10 mice. Note that the same ATPB signal is recognized by both antibodies, indicating their specificities (arrowheads). Scale bar: 10 μm. **Fig. S3** Triple immunostaining for ATPB (A, yellow), GFAP (B, red), and PDGFRβ (C, green) in the cerebral cortex of 15-week-old SAMP10 mice. Note that the ATPB signal is localized to the endfeet of GFAP+ astrocytes along the capillary, but not pericytes. Arrowheads indicate ATPB signals. Scale bar: 10 μm.

## Data Availability

The datasets supporting the conclusions of this article are available by request, but will not be posted on a repository at this point due to intellectual property/confidentiality issues.

## Abbreviations

Aβ: β-Amyloid
AD: Alzheimer’s disease
CB2: Type 2 endocannabinoid receptor
FOV: Field of view
FWHM: Full-width at half-maximum
MCI: Mild cognitive impairment
PET: Positron emission tomography
ROS: Reactive oxygen species
SAMPs: Senescence-accelerated mouse prones
SAMR1: Senescence-accelerated mouse resistant 1
SUVRs: Standard uptake value ratios
TSPO: Translocator protein 18 kD
PDGFRβ: Platelet-derived growth factor receptor-β
