# Supplementary figures and images for: In vivo alterations of mitochondrial activity and amyloidosis in early-stage senescence-accelerated mice: a positron emission tomography study
Source: J Neuroinflammation. 2021 Dec 10;18:288. doi: 10.1186/s12974-021-02343-4 (PMC8665644; doi:10.1186/s12974-021-02343-4)

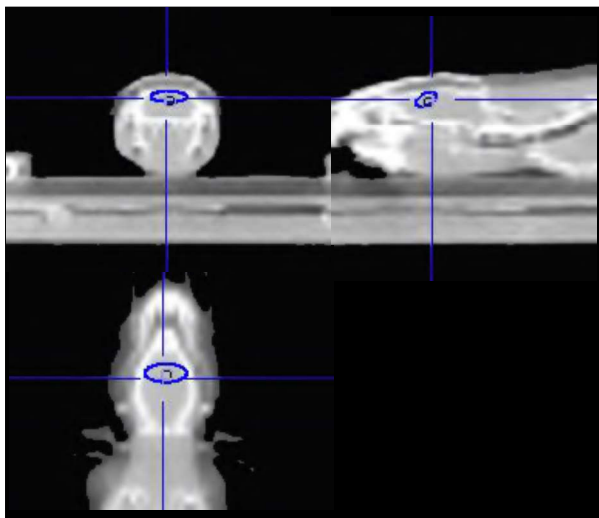

CT

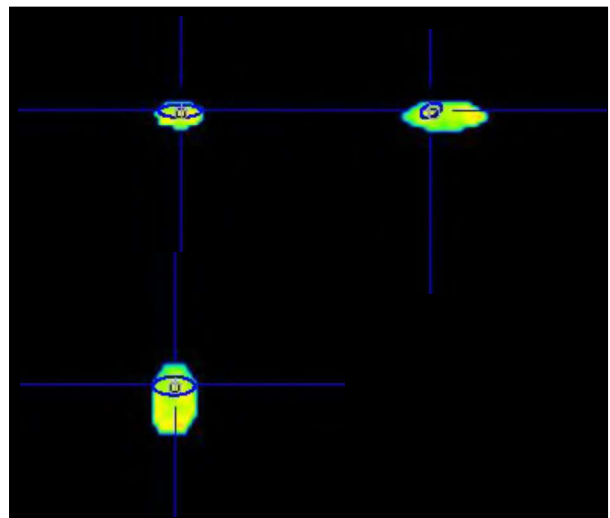

PET

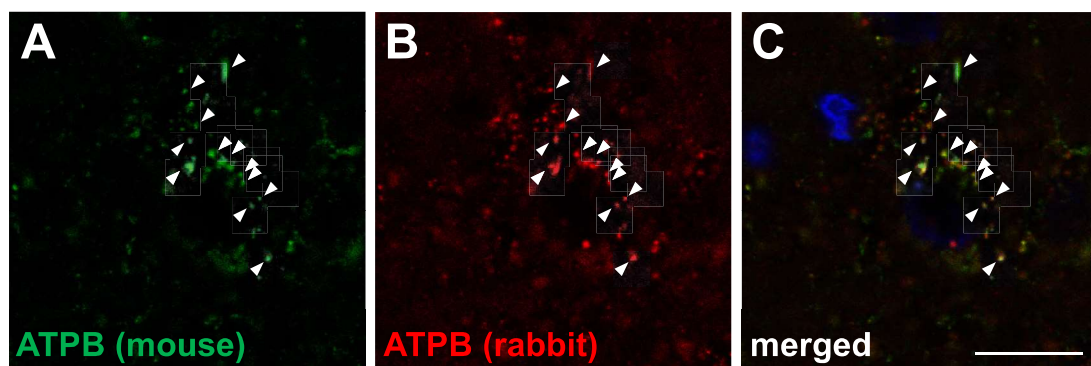

Sup Figure 2

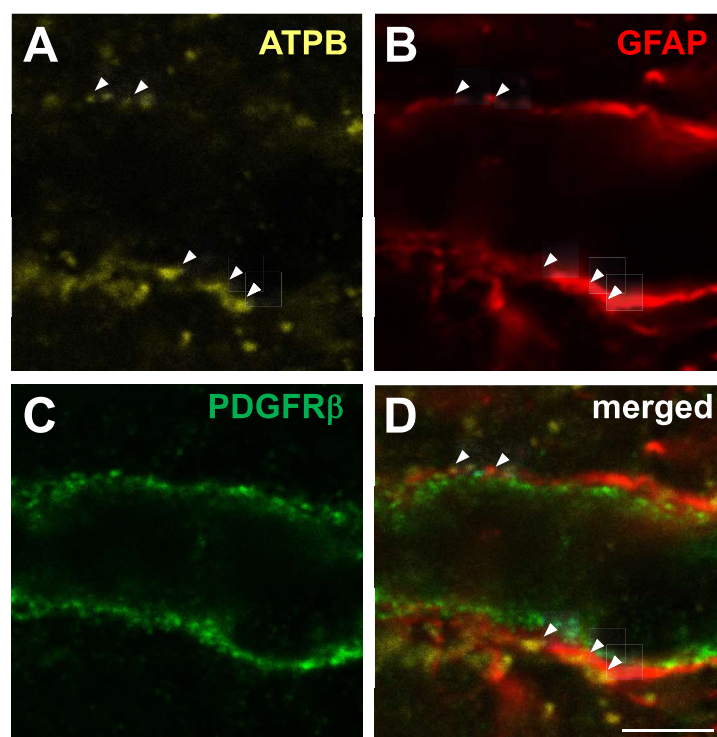

Sup Figure 3

Supplement: Supplementary file 1 — Additional file 1: Figure S1 The location of volume of interest. The VOI is displaced three-dimensionally on CT and the corresponding PET images. Fig. S2 Double immunostaining for ATPB by mouse monoclonal (A, green) and rabbit polyclonal (B, red) antibodies in the cerebral cortex of 15-week-old SAMP10 mice. Note that the same ATPB signal is recognized by both antibodies, indicating their specificities (arrowheads). Scale bar: 10 μm. Fig. S3 Triple immunostaining for ATPB (A, yellow), GFAP (B, red), and PDGFRβ (C, green) in the cerebral cortex of 15-week-old SAMP10 mice. Note that the ATPB signal is localized to the endfeet of GFAP+ astrocytes along the capillary, but not pericytes. Arrowheads indicate ATPB signals. Scale bar: 10 μm. [file 12974_2021_2343_MOESM1_ESM.pdf]
